# Real Time Observation of Lithium Insertion into Pre-Cycled Conversion-Type Materials

**DOI:** 10.3390/nano11030728

**Published:** 2021-03-14

**Authors:** Sooyeon Hwang, Dong Su

**Affiliations:** Center for Functional Nanomaterials, Brookhaven National Laboratory, Upton, NY 11973, USA; dongsu@iphy.ac.cn

**Keywords:** lithium-ion batteries, conversion, in situ TEM, lithiation

## Abstract

Conversion-type electrode materials for lithium-ion batteries experience significant structural changes during the first discharge–charge cycle, where a single particle is taken apart into a number of nanoparticles. This structural evolution may affect the following lithium insertion reactions; however, how lithiation occurs in pre-cycled electrode materials is elusive. In this work, in situ transmission electron microscopy was employed to see the lithium-induced structural and chemical evolutions in pre-cycled nickel oxide as a model system. The introduction of lithium ions induced the evolution of metallic nickel, with volume expansion as a result of a conversion reaction. After pre-cycling, the phase evolutions occurred in two separate areas almost at the same time. This is different from the first lithiation, where the phase change takes place successively, with a boundary dividing the reacted and unreacted areas. Structural changes were restricted at the areas having large amount of fluorine, implying the residuals from the decomposition of electrolytes may have hindered the electrochemical reactions. This work provides insights into phase and chemical evolutions in pre-cycled conversion-type materials, which govern electrochemical properties during operation.

## 1. Introduction

Conversion-type electrode materials (MX, where M is generally a transition metal ion and X is O, S, F, etc.) have been extensively explored, as they can deliver much higher capacities than intercalation-type materials [1,2,3,4,5,6]. When lithium ions are introduced, MX is converted into M and Li-X via the conversion reaction. From a structural point of view, MX is decomposed into a composite of M nanocrystals and Li-X matrix with the introduction of lithium ions, then the composite is rearranged into MX nanocrystals when the lithium ions are removed. Structural evolutions during the first discharge–charge cycle may change the local environments around the electrode material (strain, defects, or other microscopic factors) through volume expansion [7] and fragmentation of the bulk active material; therefore, subsequent electrochemical reactions and corresponding phase evolutions may occur in different ways than those at the initial reaction. Despite the fact that the dynamic phase evolutions at the first lithiation have been thoroughly investigated by in situ transmission electron microscopy (TEM), [8,9,10,11,12,13] the knowledge achieved from the first lithium insertion may not be applicable to understanding the structural changes in the second and following cycles. Ex situ TEM works have provided significant insights into discovering the conversion mechanisms for multiple cycles [14,15,16,17] but it is hard to understand reaction dynamics via postmortem studies. It is of importance to observe how the phase evolutions after the first cycle take place in real time since they determine the electrochemical performances during operation. However, it is still elusive how lithiation occurs in pre-cycled conversion materials.

In this work, in situ TEM was exploited to track the lithiation reaction in pre-cycled NiO as TEM is a powerful tool for investigating the physical and chemical properties of nanomaterials, and is also capable of constructing an open-cell configuration [8,9,10,11,12,13]. NiO was selected as a model system since its single redox (Ni^2+^ ↔ Ni^0^) and corresponding phase change (NiO ↔ Ni) are straightforwardly interpreted. The reconstruction of nano NiO in pre-cycled NiO resulted in different reaction behaviors during the conversion reaction. The local chemical composition also affected the occurrence of phase evolutions. This work exhibits the second lithiation reaction in conversion-type materials, which is different from the initial one and more relevant to actual battery operation.

## 2. Materials and Methods

The electrode was prepared as mixed slurries with 80 wt.% of the active NiO, 10 wt.% of carbon black, and 10 wt.% of polyvinylidene fluoride (PVDF) binder in an N-methyl pyrrolidone (NMP) solvent for an electrochemical test. The synthesis of the NiO sample was delineated in a previous report [9]. The well-mixed slurry was coated onto a Cu metal foil, which acted as a current collector. Coin cells of 2032-type were assembled with an as-prepared electrode, a Celgard separator, Li metal, and an electrolyte of 1 M LiPF_6_ in ethylene carbonate/dimethyl carbonate (DMC) (1:1 in weight) inside an Ar-filled glove box. A lacey carbon film on a copper TEM grid with dispersed NiO was also inserted into a coin cell for the forthcoming in situ TEM experiment. The coin cells were tested within a voltage range of 0.1–3 V at a rate of C/10 using galvanostatic conditions on an Arbin BT2000 battery test station (Arbin Instruments, College Station, TX, USA). After the electrochemical tests, the coin cells were disassembled inside the Ar-filled glove box and the TEM grid was thoroughly washed with DMC to remove residual salts. In situ TEM observations and analytical TEM analysis were performed with a JEM-2100F (JEOL, Tokyo, Japan) at an accelerating voltage of 200 kV. A scanning tunneling microscopy (STM)-TEM holder (Nanofactory Instruments, Göteborg, Sweden) was used to construct an open cell, where Li/Li_2_O on a piezo-driven W tip served as an anode/solid electrolyte, the lacey carbon film on the Cu TEM grid worked as the conducting agent and current collector, and cycled NiO samples were the working electrodes. During the in situ lithiation, a constant negative bias was applied to the samples to promote the movement of the Li.

## 3. Results and Discussion

**Figure 1:**
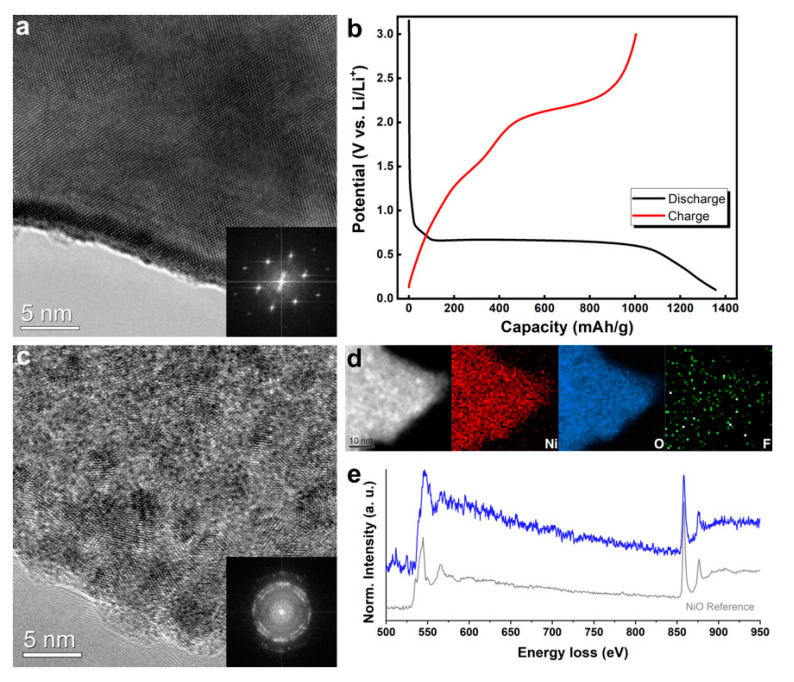
(**a**) A high-resolution TEM (HRTEM) image of pristine NiO. (**b**)The discharge–charge voltage profiles for the first cycles acquired from a coin cell. (**c**) An HRTEM image and (**d**) a high-angle annular dark-field scanning TEM (HAADF-STEM) image and the corresponding STEM- electron energy loss spectroscopy (EELS) Ni, O, and F elemental maps obtained from pre-cycled NiO. (**e**) EELS acquired from the NiO after the first cycle with that of the NiO reference.

Figure 1a presents a high-resolution transmission electron microscopy (HRTEM) image and fast Fourier transformation (FFT) result of pristine NiO. It is clear that the NiO was well-crystallized and obtained a long-range order before the electrochemical tests. After a discharge–charge cycle (Figure 1b), the microstructure of the NiO was completely changed, as shown in Figure 1c. During a discharge (lithiation), a conversion reaction occurred in the NiO (NiO + Li^+^ → Ni + Li_2_O), where Ni nanoparticles were formed with a matrix of Li_2_O [9,18]. Figure 1c suggests a charge (delithiation) took place in a way that each Ni nanoparticle became oxidized. The ring pattern of the FFT result (inset of Figure 1c) also indicates the active material consisted of a number of nanoparticles. The electron energy loss spectroscopy (EELS) elemental maps of Ni and O (Figure 1d) present the coexistence of Ni and O. The fluorine elemental map shows a trace amount of F was evenly distributed, which can be contributed to the uniform formation of a solid electrolyte interphase (SEI). The EELS spectrum of NiO after one cycle in Figure 1e is comparable with the one from the NiO reference, implying that nanocrystalline NiO was obtained after a discharge–charge cycle.

**Figure 2:**
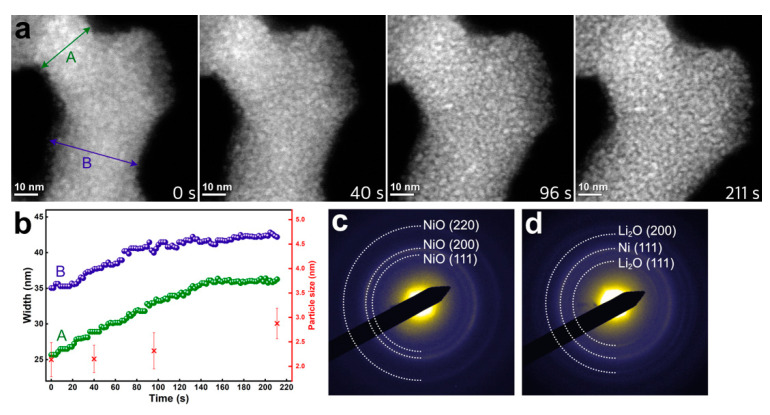
(**a**) Time-series HAADF-STEM images of pre-cycled NiO during lithiation. (**b**) The changes in the width of A and B in (**a**) and size of inside particles with lithiation. Selected area electron diffraction (SAED) patterns of (**c**) before and (**d**) after in situ lithium insertion.

The lithium insertion reaction in the pre-cycled NiO was observed in real time. An open cell was constructed inside of the TEM, as shown in previous reports [9,11,19]. Figure 2a presents a time-series of high-angle annular dark-field scanning TEM (HAADF-STEM) images during lithiation. A video showing the lithiation reaction is also provided as Video S1 in the Supplementary Materials. As lithium ions were introduced, an expansion of the active material was noticed. The changes in the widths of the pre-cycled NiO in different regions, labeled “A” and “B” (Figure 2a), were tracked for the duration of lithiation (Figure 2b), showing a continuous increase in both A and B. It is noteworthy that volume expansions were noticed in two separate areas simultaneously, indicating that the conversion reaction did not occur successively. Previous works reported the initial conversion reactions are a two-phase reaction, where a phase boundary between the reacted and unreacted areas is apparent and the phase boundary moves as lithium ions keep diffusing into the material [10,20,21,22,23]. Here, the conversion reaction started in the regions away from each other concurrently (Figure 2a,b), which may result in an S-like discharge profile during the second and following cycles, while the voltage profile shows a plateau during the initial discharge [2,24,25]. The width of the pre-cycled NiO in region A increased by 41.24%, while that in region B increased by 20.11% during observation. Considering that the NiO sample had the morphology of nanosheet [9,23], volume expansion along the z-direction was expected to be negligible. Thus, 99.26% and 44.26% of volume expansion were expected in regions A and B, respectively. Comparing the estimated volume expansion of the NiO with reference [7] suggests that most of the active materials underwent the conversion reaction around region A, while only a portion of the materials reacted with the lithium around region B. The degree of conversion reaction may differ depending on the distance from the lithium source. As lithiation proceeded, particulates in the pre-cycled NiO became larger (Figure 2b). According to the Z-contrast of the HAADF-STEM, we can conjecture that the brighter particulates in the HAADF-STEM image acquired at 211 s in Figure 2a were composed of an element with a high atomic number (Z), in this case, Ni. The bigger size of Ni particles (211 s) compared to the NiO (0 s) suggests that the metallic Ni might have been agglomerated during the conversion reaction since the unit cell of the Ni is smaller than that of the NiO. Figure 2c,d shows selected area electron diffraction (SAED) patterns acquired before and after in situ lithiation, demonstrating that the NiO was converted into Ni and Li_2_O as a result of the conversion reaction.

**Figure 3:**
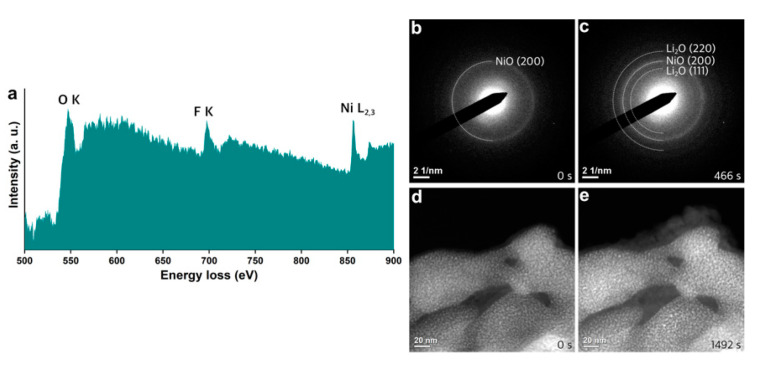
(**a**) EELS acquired from a part of pre-cycled NiO. SAED patterns and HAADF-STEM images obtained (**b**,**d**) before and (**c**,**e**) after in situ lithiation. In situ experiments were performed separately for the acquisition of SAED and HAADF-STEM.

Chemical inhomogeneity was observed in the pre-cycled NiO samples. Figure 3a shows an EELS spectrum acquired from the NiO after an electrochemical cycle, presenting an additional edge around 685 eV compared to the spectrum in Figure 1e. It was determined to be the fluorine K-edge, which can originate from the residuals of decomposed electrolytes during prior electrochemical tests in a coin cell. The SAED acquired in the area containing F (Figure 3b) indicates that the NiO was nanocrystalline, while the F-containing species were amorphous. After in situ lithiation for 466 s (Supplementary Materials, Video S2), a ring pattern from the NiO was well-kept, while ring patterns from the lithium oxide evolved (Figure 3c). This explains that the conversion reaction did not occur, though lithium ions were transported into the NiO. Diffused Li ions were transformed into Li_2_O rather than participating in the conversion reaction in the area with F, implying that the products from the decomposition of electrolytes may have limited the electrochemical reactions in the active materials. Figure 3d,e exhibits HAADF-STEM images acquired before and after in situ lithiation, where the conversion reaction did not occur, demonstrating that the formation of Li_2_O took place on the surface of the pre-cycled NiO, which is distinct from the formation of the Li_2_O through the conversion reaction shown in Figure 2. As Li_2_O was the electrical insulator, the development of Li_2_O on the surface of active material can be another obstacle for electrochemical reactions.

**Figure 4:**
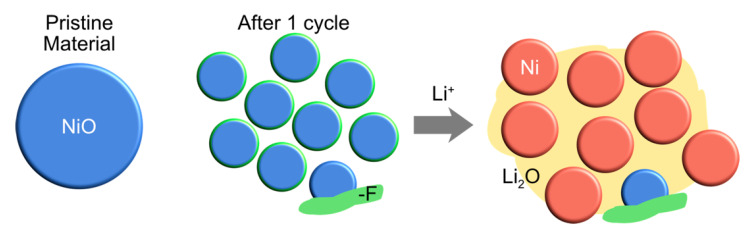
A schematic showing the structural evolution during the second lithiation.

Figure 4 summarizes findings of this work. As a result of a full discharge–charge electrochemical cycle, the whole NiO was disintegrated into a number of NiO nanoparticles. Depending on the amount of F around the NiO particles, different reaction behaviors were observed. Areas with the trace amount of F around NiO nanoparticles (green lines around NiO in Figure 4) were electrochemically active where a thin SEI layer was desirably formed. Thus, we can see the conversion reaction occurred when the lithium ions were introduced. Meanwhile, the pre-cycled NiO covered by thick F-containing species could not take part in the phase evolutions with Li ions, suggesting that products from the electrolyte decomposition may hinder the electrochemical reactions.

## 4. Conclusions

In this work, in situ electron microscopy was exploited to demonstrate the phase evolutions of pre-cycled conversion-type metal oxide materials during lithiation. The initial discharge–charge process of active materials led to converting the bulk material into a bunch of nanocrystals. Structural change may have produced the new reaction routes, reflecting that the conversion-induced volume expansion was observed simultaneously in two separate areas. S-like voltage profiles for the second and following discharge processes might have been associated with concurrent structural evolutions. Conversion reactions were not observed in the region containing substantial amount of fluorine, implying that the decomposition of electrolytes should be desirably controlled to not limit the usage of active materials. This work visualizes the lithiation reaction in pre-cycled conversion-type materials, and is related to electrochemical performances for practical operation.

## Data Availability

The data presented in this study are available on request from the corresponding author.

## Supplementary Materials

The following are available online at https://www.mdpi.com/2079-4991/11/3/728/s1: Video S1: HAADF-STEM real time movie during the lithiation reaction in the pre-cycled NiO. Accelerated 15 times. Video S2: SAED was recorded during lithiation, where a conversion reaction did not occur. Accelerated 50 times.
